# MRSA *spa* t1081, a Highly Transmissible Strain Endemic to Hong Kong, China, in the Netherlands

**DOI:** 10.3201/eid2106.141597

**Published:** 2015-06

**Authors:** Paul Gruteke, Pak-Leung Ho, Anja Haenen, Wai-U Lo, Chi-Ho Lin, Albert J. de Neeling

**Affiliations:** Onze Lieve Vrouwe Gasthuis, Amsterdam, the Netherlands (P. Gruteke);; Amsterdam Health Service, Amsterdam (P. Gruteke);; University of Hong Kong, Hong Kong, China (P.-L. Ho, W.-U. Lo, C.-H. Lin);; National Institute for Public Health and the Environment (RIVM), Bilthoven, the Netherlands (A. Haenen, A.J. de Neeling)

**Keywords:** methicillin-resistant Staphylococcus aureus, MRSA, bacteria, epidemiology, S. aureus protein A, spa typing, spa t1081, long-term care facility, hospital, the Netherlands, Hong Kong, China

**To the Editor:** Control of methicillin-resistant *Staphylococcus aureus* (MRSA) is an international public health priority. The Netherlands is among countries in Europe that have a low prevalence of MRSA among humans, largely because of a national search and destroy policy (*1*). The overall prevalence in long-term care facilities (LTCFs) is low (*2*). However, this policy is challenged by an increase in MRSA *S. aureus* protein A (*spa*) t1081, which specifically affects LTCFs. MRSA with the same *spa* type is endemic to Hong Kong, China, and affects hospitals and LTCFs (*3**–**5*). This finding prompted us to jointly explore epidemiologic and strain-related factors.

The low prevalence of MRSA enables the National Institute for Public Health and the Environment (Bilthoven, the Netherlands) to type all first MRSA isolates referred from clinical laboratories in the Netherlands. In 2007, *spa* typing replaced pulsed-field gel electrophoresis typing. The annual number of referred isolates ranged from 1,570 in 2008 to 2,439 in 2013, excluding livestock-associated strains. Numbers of MRSA *spa* t1081 isolates were low during 2007–2009 but increased to 127 isolates in 2011 and 218 isolates in 2013.

The search and destroy policy in the Netherlands requires that detection of MRSA infection is followed by screening of neighboring patients and personnel in successive circles until no new colonizations are found. Most reported t1081 isolates represent colonization. In 2013, there were 30 infection isolates, 19 unknown isolates, and 169 colonization isolates.

Severe illness caused by t1081 is rarely reported, and eradication therapy is usually successful. In LTCFs, MRSA t1081 was more prevalent, accounting for 27% (65/242) and 24% (72/299) of all MRSA isolates from LTCFs in 2011 and 2013 respectively. LTCF clusters were often small, but some became large. The t1081 strain was probably introduced into Amsterdam and subsequently spread eastward (Figure).

**Figure:**
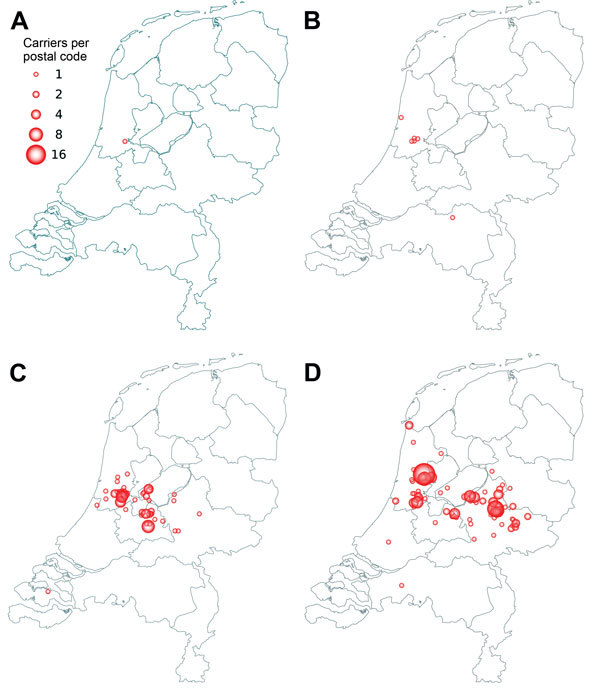
Spread of methicillin-resistant *Staphylococcus aureus* *spa* t1081 in the Netherlands, 2007–2013. A) 2007; B) 2009; C) 2011; D) 2013. Data were obtained from http://www.rivm.nl/mrsa. Source: National Institute for Public Health and the Environment (RIVM).

We sequenced 5 t1081 isolates, 3 from The Netherlands (NL2007, NL2011, and NL2013) and 2 from Hong Kong (HK2005 and HK2008), by using whole-genome sequencing (Illumina, San Diego, CA, USA). Sequence data showed the CC45/*agr* IV-MRSA V type (*3**,**6*). A total of 91.39% of reads of NL2007 aligned with the published CC45/USA600 sequence (*7*); NL2007 was more similar to HK2005 and HK2008 (97.32% and 97.61% identity, respectively). NL2011 and NL2013 showed slightly decreasing similarity to HK2005 (96.86% and 96.59% identity, respectively) and HK2008 (96.97% and 96.69% identity, respectively). Staphylococcal cassette chromosome *mec* type V sequences in our isolates were more closely related to each other than to the closest reference sequence (GenBank accession no. AB505629), which originated from a CC398 isolate.

Phenotypic resistance to tetracycline and ciprofloxacin is common in t1081 and is often combined with gentamicin and macrolide resistance. *tet*K, a gene coding resistance to tetracycline that is located on the staphylococcal cassette chromosome *mec* element, was detected in reads of all sequenced isolates. The macrolide resistance gene *erm*C on plasmid pKH19 (GenBank accession no. NC_010685.1) was detected in HK2008, NL2011, and NL2013. Resistance to gentamicin (*aac*A/*aph*D genes) was detected in all isolates. A recent report on epidemic MRSA strain 15 based on many whole-genome sequenced strains highlights antimicrobial drug use as an evolutionary driving force (*8*). The *tet*K gene might benefit t1081 in LTCFs in the Netherlands, in which doxycycline is used more frequently than in hospitals (NethMap-MARAN 2014; http://www.swab.nl/nethmap).

None of our isolates was positive for **Panton-Valentine leukocidin,** and all isolates had the collagen-binding adhesion gene. In methicillin-sensitive *S. aureus*, this gene has been associated with carriage (*9*). The apparent high transmissibility of t1081 remains to be explained.

The present t1081 outbreak has elicited a debate on the policy in the Netherlands. Some elder-care physicians question benefits and costs of this policy for a strain that is weakly pathogenic. Residents in whom MRSA carriage cannot be eradicated face prolonged measures that some physicians say are unethical. Conversely, hospital infection control professionals emphasize that if MRSA can be controlled in hospitals, why not in LTCFs? The search and destroy policy in the Netherlands faced a major challenge in 2001 (*10*). Uncontrolled dissemination of MRSA had occurred throughout a large hospital in Rotterdam among patients and staff, as well as in neighboring institutions. This outbreak was eventually controlled (*10*). The Rotterdam area, which is southwest of Amsterdam, has not been affected by the current t1081 outbreak.

Concurrent with this professional debate, a public debate is ongoing on the quality of care in LTCFs. Residents have more illnesses than a decade ago because of increasingly stringent admission criteria. Skill levels of personnel have not kept pace in several LTCFs, as noted by the Health Care Inspectorate. Although virtually all LTCFs are publicly funded, quality differences are substantial. This situation is no longer acceptable, according to public opinion. A link between insufficient skill levels in specific LTCFs and spread of MRSA can be inferred.

The latest initiative to control multidrug-resistant organisms, including MRSA in LTCFs, is included in an existing program for rapid outbreak reporting and support for hospitals by the National Institute for Public Health and the Environment. This initiative is expected to begin early in 2015 and should facilitate control of MRSA in LTCFs.
